# Resveratrol and Myopathy

**DOI:** 10.3390/nu8050254

**Published:** 2016-04-28

**Authors:** Jean Bastin, Fatima Djouadi

**Affiliations:** INSERM UMRS 1124, Université Paris Descartes, Paris 75006, France; jean.bastin@inserm.fr

**Keywords:** metabolic myopathies, mitochondrial disorders, Duchenne muscular dystrophy, resveratrol

## Abstract

Resveratrol is a natural polyphenolic compound produced by plants under various stress conditions. Resveratrol has been reported to exhibit antioxidant, anti-inflammatory, and anti-proliferative properties in mammalian cells and animal models, and might therefore exert pleiotropic beneficial effects in different pathophysiological states. More recently, resveratrol has also been shown to potentially target many mitochondrial metabolic pathways, including fatty acid β-oxidation or oxidative phosphorylation, leading to the up-regulation of the energy metabolism via signaling pathways involving PGC-1α, SIRT1, and/or AMP-kinase, which are not yet fully delineated. Some of resveratrol beneficial effects likely arise from its cellular effects in the skeletal muscle, which, surprisingly, has been given relatively little attention, compared to other target tissues. Here, we review the potential for resveratrol to ameliorate or correct mitochondrial metabolic deficiencies responsible for myopathies, due to inherited fatty acid β-oxidation or to respiratory chain defects, for which no treatment exists to date. We also review recent data supporting therapeutic effects of resveratrol in the Duchenne Muscular Dystrophy, a fatal genetic disease affecting the production of muscle dystrophin, associated to a variety of mitochondrial dysfunctions, which likely contribute to disease pathogenesis.

## 1. Introduction

Resveratrol (3,5,4′-trihydroxy-trans-stilbene) is a polyphenol that belongs to the stilbenes’ sub-group of non-flavonoids phenolic compounds, which also includes piceid, viniferins, and a variety of other compounds, *etc.* [1]. Resveratrol (RSV) is a phytoalexin, meaning that several plants naturally produce it in response to injury, or to various stress conditions such as pathogen attacks. In the human diet, resveratrol can be found in red wine and in a variety of foods including grapes, peanuts and some berries. Since its isolation for the first time in 1940 from the roots of the white Hellebore (*Veratrum grandiflorum* O. Loes) [2] and, later, in the mid-60s from the Japanese knotweed *Polygonum cuspidatum* [3], resveratrol has been studied in many different scientific fields including, at first, plant biology and nutrition. However, a renewed interest in RSV arose from the discovery of its potential cancer chemopreventive activity in 1997 [4] and, since then, reports on possible beneficial effects of resveratrol on health increased exponentially [5]. During the last decade, a large number of studies have been dedicated to the characterization of various biological properties of resveratrol, in particular its anti-proliferative, anti-inflammatory, and anti-oxidant effects. These observations suggest possible beneficial effects of resveratrol in many diseases such as cardiovascular, metabolic (diabetes, obesity), or neurodegenerative disorders (Alzheimer and Parkinson), and in different cancers [5,6]. Most of the studies supporting these hypotheses have been performed in immortalized cell lines (human or rodent) of tumoral origin, or in various vascular-, hepatic, or neuronal cellular models [7]. In comparison, much less attention has been given to the impact of resveratrol in skeletal muscle [8,9]. The first reports suggesting important effects of dietary resveratrol on energy and metabolic homeostasis were published in 2006. In these initial studies performed in high-fat fed mice receiving dietary resveratrol supplementation [10,11], the observed improvements in insulin sensitivity and the overall resistance to obesity were attributed to resveratrol-induced changes in skeletal muscle metabolism. These findings have been abundantly discussed in several excellent reviews, in the context of obesity or other common metabolic diseases, and will therefore not be addressed here. This review will rather focus on the cellular effects of RSV in the context of skeletal muscle pathologies due to genetic disorders.

## 2. Resveratrol and Metabolic Myopathies

### 2.1. Resveratrol and Mitochondrial Fatty Acid β-Oxidation Disorders

Inborn mitochondrial fatty acid oxidation (FAO) disorders encompass a large group of metabolic diseases due to mutations in one of the genes encoding the β-oxidation enzymes, resulting in impaired energy production in many tissues [12]. Clinical presentations range from hypoketotic hypoglycemia, reflecting liver failure and a low fasting tolerance in patients, to arrhythmia, cardiomyopathy and rhabdomyolysis, illustrating the key role of fatty acids as energy substrates in heart and skeletal muscle. Carnitine palmitoyl transferase 2 (CPT2) and very long chain acylCoA dehydrogenase (VLCAD) deficiencies are among the most common inherited enzyme defects of FAO, which predominantly affect skeletal muscle.

CPT2, an enzyme located in the inner mitochondrial membrane, forms a carnitine-dependent shuttle system in association with other proteins (the carnitine palmitoyl transferase 1, CPT1 and the carnitine acyl-carnitine translocase, CACT), which mediates the entry of long-chain fatty acids into the mitochondria. VLCAD, also associated with the inner mitochondrial membrane, catalyzes the first step of the Linen helix within the mitochondria, and plays a key role in the use of long-chain fatty acids as energy substrates in humans. CPT2 and VLCAD deficiencies share many common clinical features and, in particular, are associated with several possible phenotypes varying in severity, age of onset, and affected tissues. Briefly, the severe infantile form of these diseases is characterized by acute hepatic failure and life-threatening cardiomyopathy. However, the majority of patients diagnosed with CPT2 or VLCAD deficiency presents the so-called “mild” phenotype, characterized by isolated myopathy of adolescence or adult-onset. Typical manifestations include muscle stiffness and myalgia, with myoglobinuria and attacks of rhabdomyolysis, which can eventually lead to life-threatening renal complications requiring dialysis [13,14,15]. Disease symptoms can be elicited by physical exercise, but also by non-strenuous everyday life activities, or by various conditions known to stimulate fatty acid utilization (fasting, fever, exposure to cold, psychological stress, *etc*.). Exercise intolerance and myalgia lead most patients to permanently restrict their activity, with a significant impact on lifestyle and work-related activities [16,17]. As for most FAO disorders, there are to date no treatment for CPT2 or VLCAD deficiency. Management of patients is mainly supportive, based on implementation of a low-fat/high-carbohydrate diet [18,19]. However, this nutritional approach is often inefficient, and does not prevent the appearance of muscular symptoms. Accordingly, there is an urgent need for alternative therapies in order to improve the patients’ condition and quality of life. 

During the last decade, our group has explored new therapeutic strategies for CPT2 and VLCAD deficiencies, based on the use of drugs or natural compounds that might pharmacologically stimulate residual enzyme activities and β-oxidation capacities. Indeed, numerous disease-causing mutations encountered in these FAO disorders have now been identified, allowing for a better understanding of correlations between genotypes and biochemical phenotypes. To summarize, it can be said that the severe VLCAD or CPT2 gene mutations (large deletion, nonsense, truncating, but also some point mutations, *etc*.) lead to complete deficiency of the corresponding enzyme, either due to mRNA decay, to highly unstable proteins, or to fully inactive mutant enzymes. This group of mutations, responsible for a profound FAO deficiency, is generally present in patients with severe clinical presentations. Conversely, the more common “mild” missense mutations, generally associated with the muscular form of the disease, induce the production of misfolded CPT2 or VLCAD mutant proteins with detectable levels of residual enzyme activity, and palmitate oxidation capacity in the patient cells. These observations led us to hypothesize that pharmaceutical agents capable of increasing residual activity might be beneficial for correction of mild forms of CPT2 or VLCAD deficiency. This assumption was first successfully tested using bezafibrate, a widely used hypolipidemic drug, which was shown to correct FAO deficiencies *ex vivo* in CPT2 or VLCAD patients’ cells [20] through the activation of the peroxisome proliferator activated receptors (PPAR). These were the first data in support of a pharmacological correction of FAO disorder, based on a new application of the “old” drug, bezafibrate, marketed since 1986. As a matter of fact, drug repositioning has attracted growing attention, both from academic labs and from pharmaceutical industries, in the research of therapies for rare diseases in recent years [21]. 

In line with the research goal of our group, *i.e.*, to find active molecules for correction of mitochondrial diseases, we raised the hypothesis that resveratrol could enhance or correct the FAO capacities in CPT2- and VLCAD-deficient fibroblasts [22]. Palmitate oxidation rates were first measured in dose-response and time course experiments, in order to characterize the metabolic response of human fibroblasts to RSV. This and subsequent studies revealed that exposure to RSV induced a dose- and time-dependent increase in FAO capacities, already significant for 10–20 μM RSV after 24 h treatment, both in control fibroblasts and in cells from patients with the mild form of these diseases [22,23]. Panels of VLCAD- or CPT2-deficient fibroblasts with different genotypes were then routinely treated with 75 μM RSV for 48 h for maximal effects. As expected, fibroblasts from severely affected patients exhibited a profound FAO deficiency that RSV failed to improve. By contrast, in all fibroblasts from patients with the mild, myopathic form, of the diseases, RSV induced increases in FAO capacities, ranging from +50% to +360%, resulting in restoration of normal FAO levels in most cases. These data demonstrated a remarkable induction of mitochondrial FAO in response to RSV in the 11 patient cell lines tested. Correction of FAO defect was clearly related to increases in the mutated CPT2 or VLCAD protein levels in the treated cells. Since it is known that RSV is quickly metabolized in humans, we also investigated possible effects of RSV metabolites in the patient cells. Interestingly, some RSV metabolites, and in particular dihydro-RSV, one of the most abundant RSV metabolites produced by intestinal microbiota, were found to significantly stimulate residual FAO capacities in the patient cells [23]. Additionally, we tested other natural structurally related compounds of the stilbene family, and found that both of them, namely cis-RSV and trans-piceid could improve mitochondrial FAO capacities in human FAO-deficient cells [23]. This is noticeable in view of the fact that most nutrients containing RSV often contain other stilbenes. Altogether, these data therefore suggest that, in concert with RSV, some of its metabolites, and other stilbenes, might exert metabolic effects targeting mitochondrial energy metabolism in human cells. Interestingly, the marked stimulatory effects of resveratrol on FAO in patient fibroblasts is in line with observations made in rodent suggesting that resveratrol enhances FAO capacities in various immortalized cell lines, and could have similar effects *in vivo* in rats [24,25].

**Figure 1:**
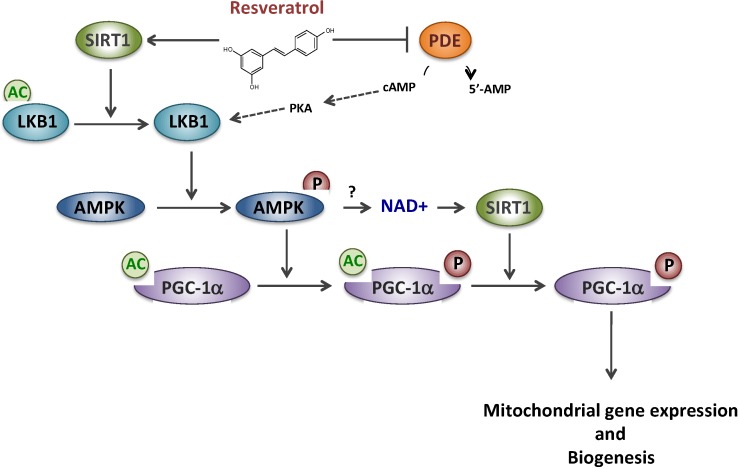
Resveratrol and putative signaling pathways involving SIRT1, AMPK and PDE. Sirtuin 1 (SIRT1), AMP-activated protein kinase (AMPK), Phosphodiesterase (PDE), cyclic AMP(cAMP), Protein kinase A (PKA), Liver linase B1 (LKB1), PPAR gamma co-activator 1 alpha (PGC-1α).

We then investigated the regulatory protein(s) involved in the signaling cascade mediating the effects of RSV. Although it is widely accepted that RSV effects are mediated through an AMPK/SIRT1/PGC-1α pathway, the molecular mechanisms and the precise order of intervention of these putative targets remain controversial [24,25]. PGC-1α is a master regulator of oxidative metabolism and mitochondrial biogenesis and its expression could be regulated both at a transcriptional or post-translational level [26]. It was first proposed by some authors that RSV would act as a potent allosteric activator of SIRT1, a member of the NAD-dependent deacetylase protein family [27], to catalyze PGC-1α deacetylation/activation [11]. Later on, this putative direct link between SIRT1 activity and RSV has, however, been questioned [28,29,30]. According to another hypothesis, RSV would first activate the AMP-activated protein kinase (AMPK), considered to be a cellular fuel gauge [10], which would then phosphorylate/activate PGC-1α [31]. Several cascades of events, summarized in Figure 1, have been proposed to reconcile both proposals: (i) a SIRT1 direct activation by RSV would lead to deacetylate/activate LKB1, a kinase that would subsequently activate AMPK by phosphorylation, and hence PGC-1α [32,33]; (ii) an indirect activation of SIRT1 would occur after a complex signaling cascade involving AMPK activation subsequent to competitive inhibition of PDE by RSV [34]. In both schemes, it is assumed that AMPK activation would ultimately lead to an increase in the NAD+/NADH ratio, inducing activation of NAD-dependent SIRT1, and the regulatory cascade would end in AMPK-mediated phosphorylation of PGC-1α that would prime for its subsequent deacetylation by SIRT1 [25].

In line with these literature data, the involvement of PGC-1α in the response to resveratrol was studied by transfection of patient fibroblasts using siRNA approaches. Disruption of PGC-1α expression abolished the induction of FAO by RSV [22]. Furthermore, treatment by RSV induced up-regulation of PGC-1α protein level, and its activation by deacetylation. In RSV-treated fibroblasts, we also observed a marked increase in gene expression and protein levels of SIRT1 [22]. Experiments using sirtinol, a compound considered as a SIRT1-specific inhibitor at that time, led us to conclude that RSV-induced stimulation of FAO in deficient fibroblasts was mostly mediated by SIRT1. However, since then, other results obtained in respiratory chain deficient fibroblasts (next paragraph), and the fact that sirtinol was shown to be a pan-sirtuin inhibitor, questioned our conclusion on a specific involvement of SIRT1 in the response to resveratrol. As mentioned above, the exact role of SIRT1 in the resveratrol signaling cascade still remains a matter of debate. We also studied the effects of two AMPK activators (AICA-riboside: 5-amino-4-imidazolecarboxamide riboside, and A-769662 compound). Under the conditions (doses/duration) used in our study, we did not reveal significant effects of these compounds on FAO in our panel of cells [22]. There is, however, a large number of drugs and natural compounds acting as direct or indirect AMPK activators that might be worth testing in order to better characterize the relevance of AMPK as a therapeutic target in FAO disorders. 

### 2.2. Resveratrol and Complex I Disorder

Dysfunctions of mitochondrial oxidative phosphorylation (OXPHOS), now described in a wide range of human pathologies, are either considered as a primary cause of the diseases, when due to mutations of mitochondrial or nuclear genome, or as secondary when associated, for example, to type II diabetes, Alzheimer, or Parkinson diseases [35]. Among the inherited mitochondrial disorders, isolated complex I (CI) deficiency is one of the most frequent [36]. In recent years, we have been particularly interested in CI disorders due to mutations in nuclear-encoded CI subunits or assembly factors. Patients with CI deficiency present with various clinical signs and symptoms including Leigh disease, a fatal neurodegenerative disorder, neonatal lactic acidosis, growth retardation, seizures, cardiomyopathy, hepatopathy, *etc*. Skeletal muscle is also frequently affected in CI deficiency, with hypotonia, dystonia and rhabdomyolysis [36]. 

As for FAO disorders, genetic and biochemical characterization of mitochondrial disorders has improved substantially in the last decades, but the therapeutic options remain very limited [37]. In recent years, however, the fine deciphering of the molecular mechanisms and signaling pathways involved in the regulation of mitochondrial energy metabolism, and in particular the identification of master regulators, has paved the way for more rational treatment strategies. Among new therapeutics hypotheses [37], our group tested the effect of RSV as a putative “booster” of mitochondrial energy production in the context of inborn CI-deficiency, based on the positive results previously obtained in inborn FAO disorders. Furthermore, numerous data in animal models suggested that activation of PGC-1α signaling cascade could mediate increases in respiratory chain capacities and mitochondrial biogenesis in tissues with high oxidative rates, such as heart or skeletal muscle [38,39].

In control fibroblasts, Western-blot analysis showed that the levels of 13 different proteins representing structural subunits or assembly factors of the five respiratory chain (RC) complexes were significantly up-regulated after cell exposure to RSV (75 μM for 48 h) [40]. In a panel of moderate CI-deficient patients’ fibroblasts, treatment by RSV was then showed to up-regulate the amount of different mutated proteins, and to significantly increase the residual CI enzyme activities in some patient cells. Additionally, our data showed that, from a functional point of view, RSV triggered an increase in cellular O_2_ consumption in the responding patient’s cells, indicating a full correction of the RC defect. Interestingly, RSV also led to the correction of a hallmark marker of CI deficiency, namely the lactate/pyruvate (L/P) ratio. Indeed, in OXPHOS-deficient patients, an increased L/P ratio, an indicator of severe lactic acidosis, is very often encountered. In parallel, investigation of the cellular mechanisms underlying the beneficial effects of RSV, using different complementary approaches, clearly revealed the induction of mitochondrial enrichment [40]. Interestingly, other studies performed in different cell systems clearly suggest that RSV stimulates energy metabolism via a stimulation of mitochondrial biogenesis, but this notion was hardly documented in human cells [24,25].

**Figure 2:**
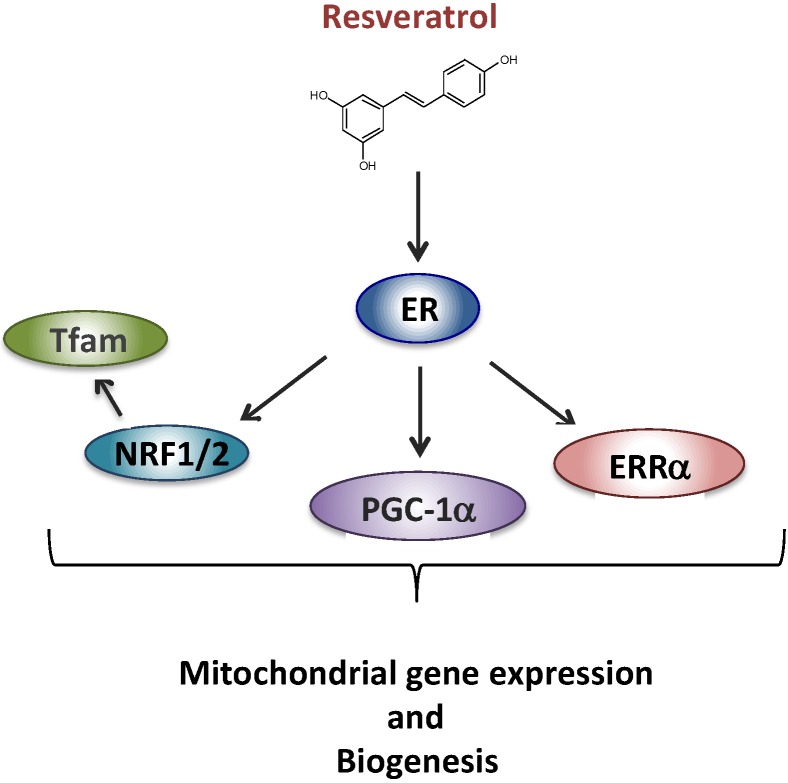
Proposed signaling pathway for the effect of resveratrol involving ER (estrogen receptor). Estrogen related receptor alpha (ERRα), PPAR gamma co-activator 1 alpha (PGC-1α), nuclear respiratory factors 1 and 2 (NRF1, NRF2), mitochondrial transcription factor A (Tfam).

We next began to unravel the signaling cascade involved in RSV effects on RC in human fibroblasts. Briefly, based on the results of siRNA experiments, we came to the conclusion that neither SIRT1 nor AMPK were involved in the signaling cascade leading to the stimulation of RC capacities in human fibroblasts, in response to RSV. These results were quite unexpected given the consensus on the role of SIRT1 and AMPK in mediating RSV effects on mitochondrial energy metabolism, established in mice and in various cell line models [24,25]. However, long before being extensively studied as a SIRT1 activator, RSV was classified as a phytoestrogen capable of binding and activating estrogen receptors (ERs) [41], which led us to focus on this signaling pathway. Based on the response to various pharmacological inhibitors, we were then able to propose a new scheme (Figure 2) to account for the metabolic response of human fibroblasts, in which RSV effects were mediated both by the ER and by the estrogen related receptor alpha (ERRα) orphan receptor [42]. 

Two other groups have recently tested the effects of RSV in RC-deficient fibroblasts. In a first study [43], the authors analyzed the response to resveratrol in fibroblasts from six patients with severe neonatal CI deficiencies. The main parameters measured were cell growth, intracellular ROS, and ATP content, after 72 h treatment by 5 μM resveratrol, or by various other small molecules, using different cell culture conditions. The response to resveratrol appeared to differ between the various CI-deficient fibroblasts, and although exhaustive data were not provided, the authors concluded on the absence of significant effects of resveratrol under these conditions. However, considering the dose-response curves established in control and CI-deficient fibroblasts in a previous study [40], it seems likely that this lack of effect was mainly due to the low dose chosen. Additionally, when using concentrations of 10 μM or lower in cultured fibroblasts, resveratrol is partially trapped on the albumin fraction of fetal calf serum [23], which reduces its cellular uptake, and hence its ability to activate intracellular targets. In another study [44], the authors investigated the effects of 100 μM resveratrol treatment for 48 h on the OXPHOS system and citrate synthase in a panel of fibroblasts from control individuals, or from patients with Complex II or complex IV deficiencies. Cell treatment by resveratrol was found to induce highly significant increases in citrate synthase, complex II, and complex IV activities in control fibroblasts, but failed to improve the deficiencies in the patient fibroblasts. According to the authors, this absence of change could be due to the severity of CII or CIV enzyme deficiency in these patients, since, in most cases, the disease-causing mutations were associated with barely detectable residual activity of the deficient complex. As pointed out by the authors, this suggests that the beneficial effects of resveratrol, or of other pharmacological treatment, are dependent on the ability of cells to produce a partially active enzyme complex, as shown in other inborn enzyme defects. 

Altogether, our data indicate that in some cells from patients with myopathic forms of RC or FAO deficiencies, treatment by resveratrol can, at least in some cases, induce pharmacological stimulation of the mutant protein level and residual enzyme activity *i.e.*, can improve the primary cause of the disorder. The results also indicate that RSV markedly induces mitochondrial fatty acid oxidation and respiratory chain capacities in control human cells, and that these beneficial effects might involve a RSV-induced stimulation of mitochondrial biogenesis, mediated by PGC-1α. 

More generally, effects of resveratrol on mitochondrial oxidative metabolism have been reported in several cells, organs and tissues and have been the topic of several reviews [5,24,25], which will not be discussed in detail here. In skeletal muscle, it has been proposed that RSV could induce positive adaptations of energy metabolism [45], and might therefore act as an exercise mimetic [46,47]. However, it should be emphasized that resveratrol was found beneficial to enhance skeletal muscle or cardiac functions in some, but not in all, rodent models tested [46,47]. Furthermore, while resveratrol may mimic certain aspects of exercise, it is still unclear whether it improves exercise performance in humans, and under which conditions (age, low- or high-intensity exercise, physiological or pathological state, *etc.*) [47]. 

## 3. Resveratrol and Duchenne Myopathy

Duchenne Muscular Dystrophy (DMD) is a fatal inherited myogenic disease caused by dystrophin deficiency, and is the most prevalent lethal X-linked myopathy [48]. Dystrophin is part of a transmembrane protein complex, which provides structural integrity to skeletal and cardiac muscle [49]. Clinically, the absence of dystrophin leads to chronic and progressive muscle weakness, exercise intolerance, followed by death by adolescence or young adulthood due to respiratory and cardiac failure. There is currently no effective therapy to slow or halt muscle degeneration in these patients [48]. The etiology of the disease is obviously linked to the primary loss of dystrophin expression, however, several other mechanisms might contribute to muscular injury, including impaired calcium homeostasis, chronic inflammation [50], and, interestingly, impaired cellular energy production and mitochondrial dysfunctions [51]. 

Over the last decades, remarkable progress has been made on understanding the pathophysiology of DMD and the mechanisms leading to muscle wasting, thanks in particular to the dystrophin-deficient mdx mouse [52]. Studies in mdx mice, and observations in DMD patients, have shown that slow oxidative muscle fibers (rich in mitochondria) are more resistant to the dystrophic pathology than the fast glycolytic fibers, therefore, suggesting that an active oxidative metabolism might represent an advantage to fight the disease [53]. The precise explanation for this situation is not known. Nevertheless, when considering therapeutic strategies, it is important to recall that DMD was initially considered as a metabolic disease, based on the plethora of energy metabolism anomalies reported in the skeletal muscle of patients, and in mdx mice as well. A recent excellent review addresses this very interesting point of view, which challenges the current opinion that DMD pathogenesis is primarily related to dys-regulation of Ca^2+^ homeostasis and, rather, proposes that DMD should be regarded as a metabolic myopathy [51]. As detailed by Timpani *et al.*, this assumption is supported by the metabolic defects in mitochondrial FAO, tricarboxilic acid cycle or respiratory chain pathways, and by the decreased ATP synthesis rates reported in the dystrophin-deficient skeletal muscle, both in animal models and in humans. On this basis, it can be thought that bioenergetics strategies aimed at restoring mitochondrial dysfunctions might be of therapeutic value in the context of DMD.

Another promising therapeutic approach for DMD, explored by different authors, aims at stimulating the expression of utrophin [54], a molecular “cousin” to dystrophin (74% similarity) present in the fetus, which is replaced by dystrophin during early development. Utrophin has been shown to functionally compensate for the absence of dystrophin, at least to some extent, when molecular or pharmacological (PPAR agonist, AICA-riboside) interventions up-regulate its expression. For example, in 2007, Handschin and co-workers [55] showed that overexpression of PGC-1α in the skeletal muscle of mdx mice increased utrophin mRNAs and ameliorated different parameters including muscle histology and running performance.

To date, activation of the SIRT1-PGC-1a axis is considered by some authors as the key-signaling pathway that would account for the beneficial effects of RSV in mdx mice including the shift in muscle fiber type, and the resistance to muscle injury and fatigue [56,57,58]. Thus, the first demonstration that RSV improves muscular dystrophy in mdx mouse was brought by Hori *et al.*, in 2011 [56]. Subsequently, different authors [57,58] tested the effects of RSV in the mdx mouse for its capacity (i) to up-regulate mitochondrial energy metabolism; (ii) to induce a shift from fast-twitch to slow-twitch fibers; (iii) to up-regulate utrophin expression. However, as suggested by some studies [59], the beneficial effects of resveratrol might also involve improvements in inflammation, oxidative stress, or Ca^2+^ homeostasis, which will not be detailed here.

The development of a cell-based high-throughput screening assay for utrophin promoter activation allowed Moowood *et al.* to screen the Prestwick chemical library of approved FDA drugs and natural compounds. Remarkably, this approach allowed establishing that resveratrol was able to up-regulate utrophin mRNAs and protein in the C2C12 rodent muscle cell line [60]. In 2012, Selsby *et al.* showed that 4 week-old mdx mice fed with RSV (100 mg/kg/day for eight weeks) showed increased resistance to fatigue but, in this study, resveratrol supply failed to improve resistance to injury, and did not significantly increase utrophin content [58]. Subsequently, using a similar dose of RSV (100 mg/kg/day for 10 days) given to five week-old mdx mice, Gordon *et al*., found that utrophin and PGC-1α gene expression were significantly increased, but without changes in the levels of the corresponding proteins [61]. Finally, in 2014, Ljubicic and colleagues [57] provided evidence that treatment of six-week-old mdx mice with the same effective dose of RSV for six weeks triggered a metabolic switch of fast (type 2) to slow (type 1) muscle fibers, clearly indicating a conversion towards an oxidative phenotype, known to be more resistant to dystrophy. This was accompanied by increases of cytochrome c oxidase subunit IV (COX IV) and of other mitochondrial energy metabolism markers, suggesting a stimulation of mitochondrial biogenesis, together with enhanced expression of myosin heavy chain specific of slow fiber types. In these experiments, a modest and not statistically significant increase in utrophin protein level was found, while a clear induction of SIRT1 and PGC-1α expression was reported [57]. These studies in mdx mice suggest that in some cases RSV might be effective in promoting induction of an oxidative myogenic program, which might lead to an improvement in the dystrophic pathology. 

## 4. Discussion and Conclusive Remarks

Taken together, these data clearly suggest that myopathies from completely different genetic origin can share unexpected common features, *i.e.*, mitochondrial dysfunctions, which are, therefore, not restricted to inborn energy metabolism defects. Indeed, skeletal muscle is characterized by a high metabolic plasticity, meaning that numerous pathophysiological states can induce adaptive changes of mitochondrial energy metabolism, often associated to variations in the fiber composition of muscle. In line with this, the beneficial effects of RSV in FAO- or RC-deficient cells, and in mdx animals as well, can partly be attributed to the ability of RSV to improve mitochondrial oxidative metabolism, and to up-regulate mitochondrial biogenesis. Interestingly, the molecular players mediating these beneficial effects, namely SIRT1, AMPK, and PGC-1α, are common in the different models of myopathies (metabolic and DMD), and this reinforces the notion that improvement of mitochondrial functions is a key element to account for RSV’s beneficial effects.

In addition to acting as a “booster” of energy metabolism, it is worth mentioning that resveratrol can also target several other cellular parameters, in particular the production of reactive oxygen species (ROS) and the inflammation status, and this might be particularly relevant in the context of mitochondrial disorders or muscular dystrophy. Indeed, excessive ROS production by deficient mitochondria has often been proposed as a key mechanism in the pathogenesis of FAO or RC disorders, and various antioxidants have been tested to mitigate the cellular consequences of mitochondrial enzyme deficiencies [62,63]. Unpublished data from our laboratory showed that several CPT2- or VLCAD- or CI-deficient cell lines exhibited levels of intracellular ROS significantly higher than those found in control cells, though to a variable extent. In all the patients’ fibroblasts, treatment with RSV resulted in the restoration of control ROS levels. In young mdx mice, Hori *et al.* found that RSV dietary supply (500 mg/kg/day for 32 weeks) significantly reduced oxidative stress and fibrosis in skeletal muscle, but did not improve inflammation, which is thought to play a critical role in muscular degeneration [56]. However, subsequent investigations by Gordon *et al.* showed that resveratrol, given to five-week old mdx mice at 100 mg/kg/day for 10 days significantly decreased total immune cell infiltration, specifically macrophage, therefore supporting marked anti-inflammatory effects of RSV in the mdx mice muscle [61]. Altogether, resveratrol is a natural compound displaying valuable biological properties that might find application in therapeutic approaches of myopathies: (i) RSV triggers increases in various mitochondrial enzyme activities (CPT2, VLCAD, CI), and up-regulates utrophin expression; (ii) RSV can alleviate oxidative stress associated with these diseases; (iii) RSV reduces inflammation. 

On the other hand, and as discussed in regards to other diseases, there are limitations to possible applications of resveratrol in the field of myopathies, which deserve to be discussed. For example, one of the debated issues is the dose of RSV and the duration of treatments that often vary among different studies, and, intriguingly, sometimes lead to markedly different conclusions. This highlights, one more time, the complexity of the molecular targets of resveratrol, and the multitude of signaling pathways that can mediate its effects, depending on the dose considered [25]. This point has recently been addressed in detail by our group [40], since we found that RSV treatment (75 μM for 48 h) stimulated mitochondrial respiratory chain via SIRT1- and AMPK-independent mechanisms in primary human fibroblasts. In contrast, using concentrations below 50 μM, several authors demonstrated a key role of SIRT1 in mediating resveratrol effects on energy metabolism in C2C12 rodent cells. A dose-dependent mechanism of action of RSV was also reported, by Ljubicic *et al*., in mdx mice [57]. Somewhat surprisingly, these authors showed that a high dose of RSV (500 mg/kg/day for 12 weeks) given to mdx mice was less effective in eliciting metabolic muscle remodeling, compared to a lower one (100 mg/kg/day for six weeks). It can be said that there is now a real awareness of the importance of the dose of RSV used to evaluate the impact of RSV on energy metabolism. 

However, it is equally important, in our view, to take into account another issue, namely the experimental model studied, since, in particular, the effects of RSV might also exhibit a marked cell-type specificity. There is obviously no perfect model to gain insights into the effects of natural dietary compounds in humans. In this regard, however, it should be noticed that, over the last decade, the vast majority of conclusions regarding potential effects of RSV were drawn from studies performed in rodents, either *ex vivo* (mainly C2C12 cells) or *in vivo* (high fat fed mice, mdx mice, *etc.*). It is clear that some discrepancies can, at least in part, be attributed to species differences between humans and rodents, as we recently highlighted with the example of bezafibrate [64]. 

Another point that warrants close attention is the pathophysiological context in which resveratrol is tested. For example, an intriguing observation is that RSV appears to potentially induce weight loss and to improve survival in high-fat fed mice [10,11], but not in mice fed a standard diet [65]. In humans, Williams *et al*. [66] and others [67] failed to find any positive effects of RSV in healthy non-obese populations, whereas others [68] reported beneficial effects of low doses of resveratrol on metabolic parameters in obese men. Altogether, a common feature of several *in vivo* studies is that resveratrol appears to preferentially exhibit effects in pathophysiological states, *i.e.*, when dysfunctions leading to various stresses exist, whereas its effects are minor or even absent in control healthy animals or humans. This is reflective of the context in which plants produce RSV and other stilbenes, *i.e.*, preferentially when they are exposed to various stresses.

Of course, if care should be taken when extrapolating data from mice to humans, care should also be taken “not to throw the baby with the water of the bath”. Indeed, the aforementioned data obtained in patient cells or in other cellular systems suggest that RSV might be an interesting candidate to improve deficient mitochondrial functions in metabolic myopathies, such FAO or RC deficiencies. Similarly, recently published data point out cellular and metabolic effects of RSV that might be relevant to slowing down degeneration in the DMD muscle. It is much too early to draw conclusions as to whether RSV could have therapeutic potential in human subjects affected by metabolic myopathies or by DMD. These findings would deserve to be tested in many clinical trials in order to develop innovative therapies for these devastating diseases. However, given the paucity of treatment available to date, it is worth seeking to identify drugs or natural compounds capable of boosting mitochondrial functions, which might find application in a number of different diseases.
